# Characterization and prevalence of ocular comorbidities and risk of legal blindness across the United States

**DOI:** 10.1038/s41433-024-03238-3

**Published:** 2024-07-31

**Authors:** Jeffrey Chu, Jacqueline K. Shaia, Neha Sharma, Matthew W. Russell, Aleksandra V. Rachitskaya, Katherine E. Talcott, Rishi P. Singh

**Affiliations:** 1 Case Western Reserve University School of Medicine, Cleveland, OH USA; 2 Center for Ophthalmic Bioinformatics, Cole Eye Institute, Cleveland Clinic, Cleveland, OH USA; 3 Cleveland Clinic Lerner College of Medicine of Case Western Reserve University, Cleveland, OH USA; 4 Cleveland Clinic Cole Eye Institute, Cleveland, OH USA; 5 Martin Hospitals, Cleveland Clinic Florida, Stuart, FL USA

**Keywords:** Epidemiology, Vision disorders

## Abstract

**Background/Objectives:**

Vision loss is a top disability in the United States (US). Patients commonly present with multiple ocular diseases, but the extent to which this places them at risk for vision loss, and if sex and race impacts this, is poorly understood. This exploratory analysis evaluated which ocular comorbidities and demographics are at highest risk for visual impairment.

**Subjects/Methods:**

A retrospective cross-sectional study was conducted through the TriNetX Analytics Network, an aggregated network encompassing over 90 million insured and uninsured patients across 50 healthcare organizations from all regions in the US. Patients with diabetic retinopathy (DR), age-related macular degeneration (AMD), retinal vein occlusion (RVO), glaucoma, and uveitis were included in this study. Ocular diseases and visual impairment were determined through ICD-10 codes. Prevalence and odds ratios were calculated while stratifying by sex and racial demographics. Statistical analyses were completed using RStudio and Excel with 95% confidence intervals calculated.

**Results:**

The comorbid conditions with the highest prevalence of visual impairment were uveitis and RVO (39.94%), uveitis and neovascular AMD (37.61%), and uveitis and glaucoma (33.23%). The comorbidity with the highest odds for visual impairment was uveitis and RVO (POR 4.86; 95% CI 4.49, 5.26). Compared to white males, Black and Hispanic males were disproportionately affected by visual impairment across ocular comorbidities.

**Conclusion:**

This study quantified the prevalence and odds of visual impairment for unilateral and comorbid ocular disease, with the addition of uveitis causing the greatest increase. Black and Hispanic males were disproportionately affected by visual impairment across comorbid conditions.

## Introduction

Vision loss ranks in the top ten disabilities for adults over 18 years of age in the United States, affecting over 7 million Americans [1, 2]. By 2050, 9 million Americans are projected to be affected by a form of visual impairment [1]. Vision loss highly impedes a patient’s quality of life including their ability to drive, read, function independently, and perform other activities of daily life [3]. As a result, impaired vision has been shown to lead to social withdrawal, family stress, and increasing mortality rates which can partially be attributed to susceptibility to daily life hazards [4].

The leading causes of vision loss in the US predominately correlate with ageing, with age-related macular degeneration (AMD), diabetic retinopathy (DR), glaucoma, and cataracts being the most prevalent [5]. Uveitis is another ocular disease that has detrimental consequences on vision especially in the working-age population while retinal vein occlusion (RVO) is the second most common cause of vision loss due to retinal vascular disease [6, 7]. Apart from cataracts, these diseases can result in irreparable damage to structures of the eye and potentially have significant consequences on vision.

The burden of these diseases is projected to dramatically increase in the coming decades, while disproportionately affecting Black and Hispanic or Latino communities [1]. Current studies have already reported a higher prevalence of DR and glaucoma among Black patients and retinal pathologies such as DR among Hispanic or Latino patients which can in part be attributed to issues of affordability, awareness of visual health, and regular access to ocular care [8–11]. In addition, NHANES reported the prevalence of diabetic retinopathy was 46% higher in Black individuals and 84% higher in Hispanic or Latino individuals compared to white individuals [12]. Emanuele et al. further corroborates this finding as they found an increased prevalence of moderate to severe DR in these underrepresented populations that could not be attributed to factors such as age, duration of diabetes diagnosis, or HbA1c levels [13]. Furthermore, the Baltimore Eye Survey, which compared primary open-angle glaucoma (POAG) among Black and white patients determined that Black individuals had a 4–5 times higher prevalence of glaucoma compared to white patients [14].

While it is known that singular conditions such as DR, AMD, glaucoma, RVO, and uveitis are notable causes of visual impairment, patients in routine clinical practice often present with several of these comorbidities making them particularly at risk for blindness or vision loss and significantly impacting their quality of life [15, 16]. One previous study from a singular academic center found an almost two-fold increase in blindness and low vision in patients with glaucoma who had comorbid retinal disease which included DR and AMD compared to glaucoma alone [17]. Mokhles et al. supports this finding as they found glaucoma patients with retinal vein occlusions (RVO), AMD, or DR to have greater visual impairments compared to glaucoma alone [18]. The consequences are significant as multiple ocular comorbidities can drastically impair quality of life. For example, patients who have both AMD and POAG were found to have a greater difficulty driving compared to patients with AMD alone or POAG alone [19].

Many studies performed to date that have examined the impact of comorbid ocular conditions on vision have focused on disease combinations with concurrent glaucoma [17–19]. In addition, one of the largest studies conducted was limited by availability of data from a single academic center that ranged from patient sample sizes of 1300–5200 [17]. However, the prevalence of visual impairment amongst patients with varying combinations of ocular diseases that do not include glaucoma remains to be examined. The advent of national aggregate electronic health record (EHR) research networks allows for examination of many ocular diseases among millions of Americans.

The purpose of this exploratory analysis was to examine the prevalence of a visual impairment in patients with multiple ocular comorbidities including DR, AMD, RVO, glaucoma, and uveitis. Additionally, ocular comorbidities were stratified by sex, race, and ethnicity to better understand which population groups are most affected by visual loss. This will allow for characterization of which combinations of ocular comorbidities incur the highest rate of visual impairment amongst various demographics in the United States.

## Materials/subjects and methods

This retrospective cross-sectional study was conducted through the TriNetX Analytics Network, a federated health research network that aggregates the de-identified EHR data of over 90 million insured and uninsured patients across 50 healthcare organizations (HCOs) from all geographic regions in the United States. Each HCO had on average 10–12 years of EHR data at the time of the study. Data were collected over 8 weeks from August to October 2022. TriNetX, LLC is compliant with the Health Insurance Portability and Accountability Act (HIPAA), the US federal law which protects the privacy and security of healthcare data, and any additional data privacy regulations applicable to the contributing HCO. The process by which the data is de-identified is attested to through a formal determination by a qualified expert as defined in Section §164.514(b)(1) of the HIPAA Privacy Rule. This process also deemed this study exempt from the Western Institutional Review Board approval.

Patients with DR, neovascular AMD, non-neovascular AMD, RVO, glaucoma, and uveitis were included in this study and were evaluated individually and in combination with each other. These diseases were chosen as they are established leading causes of blindness. Demographic information including gender, race, and ethnicity were collected. All ages were included in this study. ICD codes were used to extract all data regarding ocular diseases and visual impairment from TriNetX. The primary outcome measure of visual impairment was defined by blindness and low vision and determined via the ICD-10 code, H54. Additional information regarding ICD-10 codes were listed in Supplementary Table 1.

The prevalence of disease and combinations of disease were calculated from a singular time point within the 3-month collection period and compared to the entire TriNetX cohort to utilize the same control population for each disease state. This allowed for singular diseases and comorbid diseases to be compared to each other. Prevalence was reported as the number of individuals with the disease or diseases per 100,000 individuals in the TriNetX database. For example, to calculate the DR prevalence, we divided the total number of patients with DR by the total TriNetX population and multiplied this value by 100,000 to determine the prevalence per 100,000 individuals. Additionally, the percent with a visual impairment within each disease was calculated for singular disease and combinations of disease. As this was a cross-sectional study where the duration of disease was not determined, crude prevalence odds ratios (POR) of visual impairment in singular and comorbid ocular diseases were calculated using single logistic regression analysis and further stratified according to race and sex demographics [20]. The POR was calculated from a singular time point within the 3-month collection period using the equation for odds ratio POR = (A/C)/(B/D), with A/C representing the odds that a case was exposed and B/D representing the odds that a control was exposed. The control served as a reference group for comparison. For example, the POR of singular disease were compared against the total population, while the POR of comorbid disease was compared against a singular disease to provide insight on the potential impact of a comorbid condition on visual impairment. The POR of visual impairment across race and sex demographics utilized the white male as the reference group to assess Black, Hispanic or Latino, Asian, and female patient cohorts. Statistical analyses were completed using RStudio (2021.09.0) and Microsoft Excel and utilized 95% confidence intervals.

## Results

The TriNetX US Collaborative Network database had a total population of 90,380,108 at the time of data extraction. Basic demographic information regarding ocular diseases which included number of patients, average age, sex, race, and ethnicity were listed in Supplementary Table 2. Among the conditions studied, glaucoma was most prevalent ocular diagnosis within the entire TriNetX cohort at 732,324 patients (810 per 100,000) and RVO had the lowest prevalence in the population at 67,669 patients (75 per 100,000) (Table 1). The prevalence of having a visual impairment which is composed of the sum of patients with low vision and blindness was highest among neovascular AMD patients at 21.24% and lowest among DR patients at 12.63% (Table 1).

**Table 1:** Prevalence of visual impairment in singular ocular diseases from TriNetX.

Disease	Number with disease age 40 + (per 100,000)	Number with disease all ages (per 100,000)	Percent with VI within disease all ages
DR	426,497 (831)	443,105 (490)	12.63
Glaucoma	708,244 (1379)	732,324 (810)	14.73
Uveitis	124,584 (243)	167,372 (185)	12.64
Neovascular AMD	92,465 (180)	92,849 (103)	21.24
Non-neovascular AMD	199,515 (389)	200,140 (221)	15.28
RVO	66,267 (129)	67,669 (75)	17.76

Total TriNetX Population: 90,380,108.

TriNetX Population Age 40+: 51,345,226.

*DR* diabetic retinopathy, *RVO* retinal vein occlusion, *AMD* age-related macular degeneration, *VI* visual impairment.

The comorbid ocular condition pair that had the highest prevalence in the TriNetX population was DR and glaucoma at 63,667 patients (70 per 100,000) and the lowest prevalence was uveitis and RVO at 2654 patients (3 per 100,000). Although uveitis and RVO had the lowest prevalence in this population, this combination had the highest percent of patients with a visual impairment at 39.94%. The second and third disease combinations with the highest prevalence of visual impairment also included uveitis with uveitis and neovascular AMD at 37.61% and uveitis and glaucoma at 33.23%. DR and non-neovascular AMD had the lowest percent of patients with a visual impairment at 20.49%. Remaining prevalence data can be found in Table 2.

**Table 2:** Prevalence of visual impairment in ocular comorbidities from TriNetX.

Disease	Number with disease (per 100,000)	Number of VI within disease (%)
DR + Glaucoma	63,667 (70)	19,507 (30.64)
DR + Uveitis	11,444 (13)	3226 (28.19)
DR + RVO	11,204 (12)	2744 (24.49)
DR + Neovascular AMD	10,222 (11)	2792 (27.31)
DR + Non-neovascular AMD	17,963 (20)	3680 (20.49)
Glaucoma + Uveitis	32,868 (36)	10,921 (33.23)
Glaucoma + RVO	17,396 (19)	5423 (31.17)
Glaucoma + Neovascular AMD	17,670 (20)	5668 (32.08)
Glaucoma + Non-neovascular AMD	36,775 (41)	9001 (24.48)
Uveitis + RVO	2654 (3)	1060 (39.94)
Uveitis + Neovascular AMD	3201 (4)	1204 (37.61)
Uveitis + Non-neovascular AMD	5889 (7)	1806 (30.67)
RVO + Neovascular AMD	4413 (5)	1258 (28.51)
RVO + Non-neovascular AMD	7425 (8)	1848 (24.89)

Total TriNetX Population: 90,380,108.

*DR* diabetic retinopathy, *RVO* retinal vein occlusion, *AMD* age-related macular degeneration, *VI* visual impairment.

**Fig. 1:**
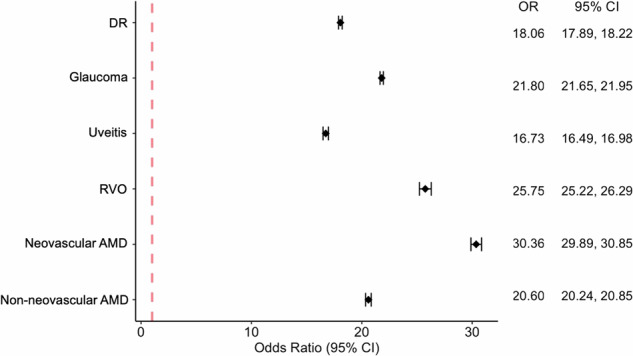
Prevalence odds ratios of visual impairment for singular ocular diseases compared to general population from TriNetX. DR diabetic retinopathy, RVO retinal vein occlusion, AMD age-related macular degeneration, OR odds ratio, CI confidence interval.

**Fig. 2:**
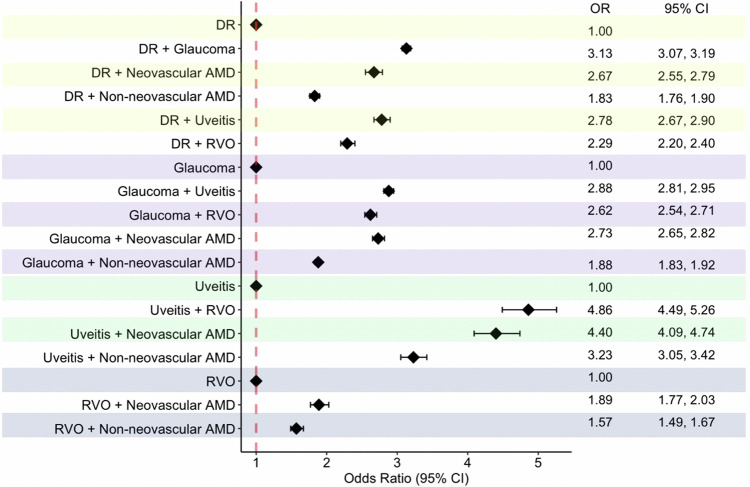
Prevalence odds ratios of visual impairment for ocular comorbidities compared to singular ocular disease from TriNetX. DR diabetic retinopathy, RVO retinal vein occlusion, AMD age-related macular degeneration, OR odds ratio, CI confidence interval.

In regard to singular ocular conditions, neovascular AMD had the largest POR for visual impairment at 30.36 times the odds compared to the total TriNetX Population (95% CI 29.89, 30.85) (Fig. 1). The singular ocular condition with the smallest POR for visual impairment was uveitis at 16.73 (95% CI 16.49, 16.98). Remaining POR data regarding singular ocular disease can be found in Fig. 1. To better understand the extent a comorbid ocular condition contributes to visual impairment, each comorbid condition had one of the singular diseases as a reference group. Regardless of combinations, each comorbid condition had an increased POR for visual impairment compared to the selected reference group. Despite having the lowest POR for visual impairment among the singular ocular diseases, disease combinations with uveitis had the largest PORs for visual impairment. Specifically, the ocular disease combination with the largest POR for visual impairment was uveitis and RVO at 4.86 times the odds compared to uveitis alone (95% CI 4.49, 5.26), followed by uveitis and neovascular AMD at 4.40 times the odds (95% CI 4.09, 4.74) and uveitis and non-neovascular AMD at 3.23 times the odds (95% CI 3.05, 3.42) (Fig. 2). Remaining POR data regarding comorbid ocular disease can be found in Fig. 2.

### Differences between race and sex

Across race and sex demographics, Black and Hispanic males were most disproportionately affected by visual impairment across the majority of comorbidities. Black males had higher POR for visual impairment compared to white males in DR and uveitis, DR and RVO, glaucoma and RVO, RVO and neovascular AMD, and RVO and non-neovascular AMD. Hispanic males had higher POR for visual impairment compared to white males in DR and glaucoma, DR and RVO, DR and non-neovascular AMD, glaucoma and uveitis, glaucoma and RVO, and glaucoma and non-neovascular AMD. White females, Asian males and females had comparable or had lower POR compared to white males for most disease combinations (Table 3).

**Table 3:**
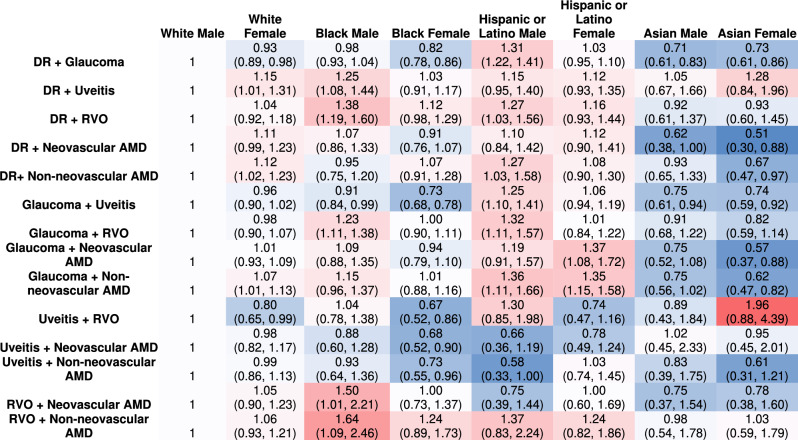
Prevalence odds ratios of visual impairment across race and sex from TriNetX.

Red represents prevalence odds ratios greater than 1 and blue represents prevalence odds ratios less than 1. Intensity of color represents distance from white male baseline of 1. 95% confidence interval reported under POR.

*DR* diabetic retinopathy, *RVO* retinal vein occlusion, *AMD* age-related macular degeneration, *POR* prevalence odds ratio.

## Discussion

This study evaluated millions of patients who were diagnosed with ocular diseases that are high causes of visual impairment in the US. In summary, we found that the most prevalent singular and comorbid ocular diagnoses across all ages were glaucoma at 732,324 patients (810 per 100,000) and DR and glaucoma at 63,667 patients (70 per 100,000). The singular ocular diagnosis with the highest prevalence and POR of visual impairment compared to the entire TriNetX population was neovascular AMD at 21.24% and 30.36 respectively. The comorbid ocular diagnoses with the highest prevalence of visual impairment all included uveitis with the combination of uveitis and RVO being the largest at 39.94%. Across comorbid ocular diseases, disease combinations with uveitis had the highest POR of visual impairment compared to a singular disease reference group with uveitis and RVO having the largest POR of 4.86. Lastly, Black and Hispanic males were most disproportionately affected by visual impairment across the majority of comorbid ocular conditions.

Overall, glaucoma had the highest prevalence in the TriNetX population while RVO had the smallest prevalence. In comparison to the literature, we did find that our glaucoma and RVO prevalence was lower [6, 21]. While some sources indicate that among the diseases studied, DR is the most prevalent condition in the US population, our study found glaucoma to be most prevalent [22]. The Vision and Eye Health Surveillance System (VEHSS) supports our finding as they also determined glaucoma to have the highest prevalence and RVO to have the lowest [23]. The calculated uveitis prevalence of 0.19% parallels the findings of other studies that have reported a prevalence of 0.13% [24]. Lastly, our neovascular and non-neovascular AMD prevalence findings confirm previous literature reports of a higher proportion of non-neovascular AMD compared to neovascular AMD [25, 26].

We consistently found neovascular AMD to have the highest prevalence and POR for having any visual impairment. This is corroborated by the literature in which multiple studies have reported that AMD, specifically neovascular AMD, is the most common cause of irreversible vision loss as it is associated with rapid disease progression [27–29]. Our findings also report that within patients with DR, 12.63% have some form of a visual impairment which is supported by Lundeen et al. who found a similar visual impairment prevalence of 19.17% in DR patients. This variability may be explained by underutilization of ICD coding in daily clinical practice [30, 31]. Lastly, our findings corroborate the Beaver Dam Eye Study in which Klein et al. found that age was a significant predictor of decreased visual acuity [32]. While we did not directly ascertain the prevalence of visual impairment in the above 40 years old population, the increased rate can be inferred from the average age data in Supplementary Table 2 which demonstrates that each visually impairing condition investigated impacts patients later in life.

In regard to comorbid ocular disease, disease combinations with uveitis had the highest prevalence of visual impairment with uveitis and RVO at 39.94%. While the literature does not report any direct findings regarding the high prevalence of visual impairment in ocular comorbidities with uveitis, it can be hypothesized that the inflammatory nature of uveitis may exacerbate RVO, AMD, and glaucoma and increase the severity and prevalence of vision loss. Because retinal inflammation from uveitis may result in damage to the vascular endothelium, this may activate the coagulation pathway, predisposing individuals to more frequent RVO events [33]. The local inflammation induced by uveitis could also lead to migration of vascular endothelial cells by cytokines, resulting in increased angiogenesis and further progression of neovascular AMD [34]. Lastly, uveitis has been known to drive and potentially worsen glaucoma in the form of uveitic glaucoma as inflammatory cells can enter the anterior chamber and disrupt aqueous outflow equilibrium [35]. These mechanisms shed light on how uveitis as a comorbidity might result in a greater risk of visual impairment.

The disease combination of DR and non-neovascular AMD was found to have the lowest prevalence of a visual impairment. This aligns with our current understanding as DR had the lowest prevalence of visual impairment and that both DR and non-neovascular AMD do not typically cause significant visual impairment until advanced disease states [36, 37]. The presence of AMD may also be protective against the development of DR and decrease the severity, as the presence of AMD alters outer retina metabolism and affects oxygen demand of the retina [38]. Furthermore, our study found that glaucoma with the addition of a retinal comorbidity had a visual impairment prevalence ranging from 24.48% to 32.08% while the visual impairment for glaucoma alone was 14.73%. This approximate 2-fold increase in visual impairment in patients with glaucoma and a retinal comorbidity compared to glaucoma alone was also reported by Griffith et al. [17]. Our findings concretely support Griffith et al.’s findings across multiple ocular disease combinations. Lastly, while Klein et al. in the Wisconsin Epidemiology Study of Diabetic Retinopathy did not find that comorbid DR and glaucoma had an increased risk of visual impairment compared to DR alone, our study found both an increased prevalence and odds of visual impairment in these patients [39]. This is likely due to the small sample size, as only 0.8% of 482 patients in their study had comorbid DR and glaucoma [39].

Our study found both Black and Hispanic males had the most significant increase in prevalence odds for visual impairment across comorbidities compared to white males. This is reflected in the literature with studies reporting an increased prevalence and odds of visual impairment in Mexican American and non-Hispanic black individuals compared to non-Hispanic white individuals [2, 40, 41]. From the Baltimore Eye Survey, Tielsch et al. further support our racial disparity findings, as they found that the overall age-adjusted rates of blindness and visual impairment among Blacks were twice those of whites [42]. However, our study not only demonstrates that this disparity extends to comorbid ocular conditions, but also quantifies the increased risk of visual impairment in minority populations. Furthermore, we found that both Asian males and females and white females had similar or lower prevalence odds of visual impairment compared to white males. Previous studies support our findings on the similarity in visual impairment levels between the white and Asian populations [43].

Lower quality of life is a sequalae of vision loss that has been well established throughout the literature [44]. In our study, we found that patients with comorbid ocular diseases had both an increased prevalence and prevalence odds of visual impairment compared to singular ocular diseases. As a result, this patient cohort is at an even higher risk for the complications of vision loss which include increased reports of life dissatisfaction, poorer reports of overall physical and mental health, and limited days of physical activity [45]. In addition, this increase in vision loss can further complicate the management of chronic conditions such as diabetes and glaucoma as patients are less capable of administering medications and attending physician appointments [3].

Although TriNetX provides our study with an extremely large sample size, it is a retrospective cross-sectional database and is not without limitations. First, and most importantly, this study relied on ICD-10 codes to obtain patients who had the diagnosis of the specific ocular condition and visual impairment status. Depending on the provider and the health system, coding patterns can vary drastically. As a result, visual impairment diagnoses based on ICD codes for low vision or blindness, which may not be consistently used in practice, could likely be an underestimation of the true extent of visual impairments which may be more reflective in survey ascertained studies. Because this study was an exploratory analysis within the entire TriNetX population, we did not stratify by age for the visual impairment analysis. However, the lack of age stratification is not meant to reflect that the conditions are evenly distributed across the age spectrum as they predominately affect middle age and older adults as seen in Table 1 and Supplementary Table 2. In addition, we were unable to obtain temporality in this analysis. Lastly, the examination of visual impairment by race and sex differences did not account for age differences and other forms of granularity within the population.

This exploratory study evaluated a large cohort of Americans with ocular disease and the prevalence of visual impairment among these diseases. We found that the addition of a comorbid ocular disease resulted in increased prevalence and prevalence odds of visual impairment across all ocular conditions. Specifically, the addition of uveitis as a comorbidity appeared to cause the greatest increase in visual impairment. Hispanic and Black males are also disproportionately affected by visual impairment across ocular comorbidities compared to white males. While it has already been established that singular ocular diseases cause vision loss, our study was able to quantify the extent and risk of vision loss in comorbid ocular disease. These findings highlight the need to monitor minority patients for visual impairment and those with a comorbid ocular condition, particularly uveitis.

## Summary

### What was known before

- Persons with ocular disease may develop additional ocular conditions.

### What this study adds

- Quantifies the prevalence of vision loss within common comorbid ocular diseases.

## Supplementary information

Supplemental Table 1

Supplemental Table 2

## Data Availability

All data generated or analyzed during this study are included in this published article.
