# Supplementary material for: Characterization and prevalence of ocular comorbidities and risk of legal blindness across the United States
Source: Eye (Lond). 2024 Jul 31;38(16):3118–24. doi: 10.1038/s41433-024-03238-3 (PMC11543832; doi:10.1038/s41433-024-03238-3)
Supplement: Supplementary file 1 — Supplemental Table 1 [file 41433_2024_3238_MOESM1_ESM.docx]

**Supplemental Table 1: ICD-10 Codes**

| **ICD-10 Code** | **Diagnosis** |
| --- | --- |
| **H40.1, H40.2, H40.3, H40.4, H40.5, H40.6, H40.8, H40.9, H42** | Glaucoma |
| **E10.31-10.35, E11.31-11.35** | Diabetic retinopathy |
| **H35.31** | Non-neovascular age-related macular degeneration |
| **H35.32** | Neovascular age-related macular degeneration |
| **H34.81, H34.83** | Retinal vein occlusion |
| **H20, H30.02, H30.03, H30.2, H30.9, H44.0, H44.01, H44.11** | Uveitis |
| **H54** | Blindness and low vision |
