# Supplementary material for: Characterization and prevalence of ocular comorbidities and risk of legal blindness across the United States
Source: Eye (Lond). 2024 Jul 31;38(16):3118–24. doi: 10.1038/s41433-024-03238-3 (PMC11543832; doi:10.1038/s41433-024-03238-3)
Supplement: Supplementary file 2 — Supplemental Table 2 [file 41433_2024_3238_MOESM2_ESM.docx]

**Supplemental Table 2:** Demographic table for singular ocular diseases from TriNetX.

^a^DR = diabetic retinopathy; RVO = retinal vein occlusion; AMD = age-related macular degeneration

^b^Total TriNetX Population: 90,380,108

| **Disease** | **None** | **DR** | **Glaucoma** | **Uveitis** | **Neovascular AMD** | **Non-neovascular AMD** | **RVO** |
| --- | --- | --- | --- | --- | --- | --- | --- |
| **Number of Patients (total)** | 88,572,506 | 443,105 | 732,324 | 167,372 | 92,849 | 200,140 | 67,669 |
| **Number of Patients (40+)** | 56,936,825 | 426,497 | 708,244 | 124,584 | 92,465 | 199,515 | 66,267 |
| **Average Age (sd)** | 46 (25) | 68 (14) | 75 (15) | 55 (22) | 83 (9) | 82 (9) | 75 (14) |
| **Male (%)** | 46 | 49 | 44 | 45 | 37 | 36 | 46 |
| **Female (%)** | 53 | 49 | 54 | 54 | 59 | 58 | 51 |
| **White (%)** | 56 | 52 | 56 | 51 | 73 | 67 | 61 |
| **Black (%)** | 13 | 20 | 20 | 25 | 3 | 3 | 14 |
| **Asian (%)** | 3 | 3 | 4 | 3 | 2 | 2 | 3 |
| **Hispanic or Latino (%)** | 10 | 13 | 6 | 8 | 2 | 3 | 6 |
